# 3-Diethyl­carbamoyl-2′,4′-difluoro­biphenyl-4-yl 2,6-dichloro-5-fluoro­pyridine-3-carboxyl­ate

**DOI:** 10.1107/S1600536812025354

**Published:** 2012-06-13

**Authors:** Chun-nian Xia, Yu Zhou, You-bao Chen, Guang-xiang Zhong

**Affiliations:** a College of Pharmaceutical Science, Zhejiang University of Technology, Hangzhou, 310032, People’s Republic of China

## Abstract

In the title compound, C_23_H_17_Cl_2_F_3_N_2_O_3_, the mol­ecular conformation is significantly strained: atoms O, C(=O) and C attached to the central benzene ring deviate from its plane by 0.118 (2), 0.139 (2) and 0.174 (2) Å, respectively. In the crystal, weak C—H⋯O inter­actions link the mol­ecules into chains along [110]. The crystal packing exhibits short inter­molecular Cl⋯F [2.9840 (16) Å] and Cl⋯Cl [3.2957 (12) Å] contacts.

## Related literature

For details of the synthesis, see: Zhong *et al.* (2009 ▶, 2010 ▶).
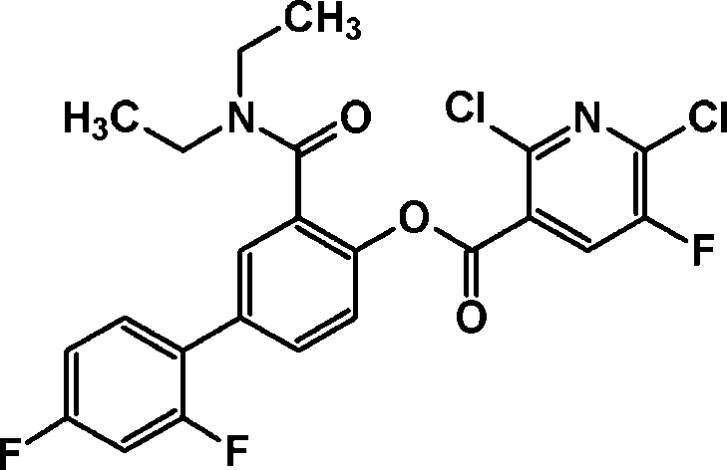


## Experimental

### 

#### Crystal data

C_23_H_17_Cl_2_F_3_N_2_O_3_
*M* *_r_* = 497.29
Triclinic, 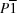

*a* = 10.635 (3) Å
*b* = 10.888 (3) Å
*c* = 11.310 (3) Å
α = 96.838 (1)°
β = 109.213 (1)°
γ = 116.017 (3)°
*V* = 1056.5 (5) Å^3^
*Z* = 2
Mo *K*α radiation
μ = 0.36 mm^−1^
*T* = 153 K
0.47 × 0.45 × 0.41 mm

#### Data collection

Rigaku AFC10/Saturn724+ diffractometer
Absorption correction: multi-scan (*SADABS*; Sheldrick, 1996 ▶) *T*_min_ = 0.848, *T*_max_ = 0.866
10358 measured reflections
4752 independent reflections
3984 reflections with *I* > 2σ(*I*)
*R*_int_ = 0.021

#### Refinement

*R*[*F* ^2^ > 2σ(*F* ^2^)] = 0.034
*wR*(*F* ^2^) = 0.082
*S* = 1.00
4752 reflections
300 parameters
H-atom parameters constrained
Δρ_max_ = 0.48 e Å^−3^
Δρ_min_ = −0.26 e Å^−3^

### 

Data collection: *CrystalClear* (Rigaku/MSC, 2008 ▶); cell refinement: *CrystalClear*; data reduction: *CrystalClear*; program(s) used to solve structure: *SHELXS97* (Sheldrick, 2008 ▶); program(s) used to refine structure: *SHELXL97* (Sheldrick, 2008 ▶); molecular graphics: *SHELXTL* (Sheldrick, 2008 ▶); software used to prepare material for publication: *publCIF* (Westrip, 2010 ▶) and *PLATON* (Spek, 2009 ▶).

## Supplementary Material

Crystal structure: contains datablock(s) global, I. DOI: 10.1107/S1600536812025354/cv5305sup1.cif

Structure factors: contains datablock(s) I. DOI: 10.1107/S1600536812025354/cv5305Isup2.hkl

Supplementary material file. DOI: 10.1107/S1600536812025354/cv5305Isup3.cml

Additional supplementary materials:  crystallographic information; 3D view; checkCIF report

## Figures and Tables

**Table 1:** Hydrogen-bond geometry (Å, °)

*D*—H⋯*A*	*D*—H	H⋯*A*	*D*⋯*A*	*D*—H⋯*A*
C2—H2⋯O3^i^	0.95	2.35	3.287 (2)	170

Symmetry code: (i) 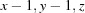.

**Fig. 1.**
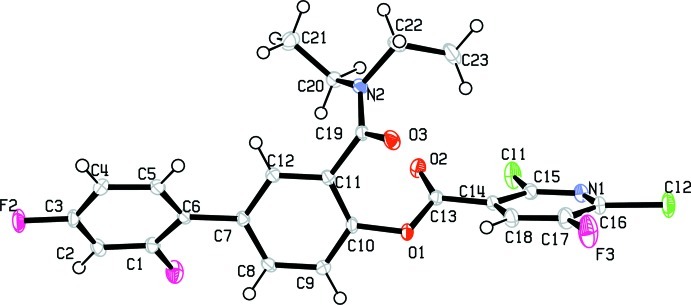
The molecular structure of (I) showing the atomic numbering and 50% probability displacement ellipsoids.

## supplementary crystallographic information

### Comment

Fluorine-containing drugs, such as tegadifur, flutamide, ciprofloxacin - non-steroid anti-inflammatory drugs, have been studied due to their special properties (Zhong *et al.*, 2009, 2010). As a continuation of their study, we present here the title compound (I) (Fig. 1).

The molecular conformation of (I) is significantly strained - atoms O1, C19 and C6 attached to the central benzene ring C7—C12 deviate from its plane at 0.118 (2), 0.139 (2) and 0.174 (2) Å, respectively. The atoms of C1–C6, F1 and F2 are almost coplanar, deviating from the mean plane within 0.0249 (12) A °. The atoms C13–C18, N1, F3 and Cl1 are coplanar, deviating from the mean plane within 0.0442 (10) A °, and the deviation of Cl1, O2 from the plane is 0.1047 (15) A °, 0.5851 (17) A °, respectively. The diethylamine group shows a normal twist conformation. The mean planes of C1—C6/F1/F2 and C13—C18/N1/F3/Cl1 form the dihedral angles of 34.62 (5) and 87.45 (4)°, respectively, with the central benzene ring. The adjacent substituent (C10 and C11 of the parent ring) remove away or reverse each other, which owes to the Cl and F atoms showing greater repulsive force. This phenomenon is samilar to that observed in 20,40-difluoro-4-[(4-chlorobenzoyl)oxy]-*N*-[4-nitro-3- (trifluoromethyl)phenyl]-[1,10-biphenyl]-3-carboxamide (Zhong *et al.*, 2010).

In the crystal, weak C—H···O interactions (Table 1) link the molecules related by translation in [110] into chains. The crystal packing exhibits short intermolecular Cl···F [2.9840 (16) Å] and Cl···Cl [3.2957 (12) Å] contacts.

### Experimental

The title compound was synthesized according to the known methods (Zhong *et al.*, 2009, 2010). M.p. 125-127°C. ^1^HNMR (500 MHz, CDCl_3_, δ ppm): 1.13 (t, 3H, *J* =7.0 Hz, -CH_3_), 1.15 (t, 3H, *J* =7.0 Hz, -CH_3_), 3.31 (q, 2H, *J*=7.0 Hz, -CH_2_), 3.50 (q, 2H, *J*=7.0 Hz, -CH_2_), 6.95 (t, 1H, *J*=8.5 Hz), 7.00 (t, 1H, *J*=8.5 Hz), 7.41(d, 1H, *J*=8.5 Hz), 7.42 (q, 1H, *J*=8.5 Hz), 7.50 (s, 1H), 7.60 (d, 1H, *J*=8.5 Hz), 8.24 (d, 1H, *J*=7.0 Hz). MS: m/z 496 (M^+^, 11.85), 425 (6.50), 233 (22.78),232 (7.63), 195 (6.76), 191 (62.99), 164 (4.63), 72 (23.72). The solid product was dissolved in butanone-ethanol (4:1 v/v) and the solution evaporated gradually at room temperature to afford colorless single crystals of (I).

### Refinement

Methyl H atoms were placed in calculated positions, with C—H = 0.96 Å, and torsion angles were refined to fit the electron density [*U*_iso_(H) = 1.5*U*_eq_(C)]. Other H atoms were placed in calculated positions, with C—H = 0.93 Å, and refined in riding mode, with *U*_iso_(H) = 1.2*U*_eq_(C).

### Crystal data

**Table:**

C_23_H_17_Cl_2_F_3_N_2_O_3_	*Z* = 2
*M**_r_* = 497.29	*F*(000) = 508
Triclinic, *P*1	*D*_x_ = 1.563 Mg m^−^^3^
Hall symbol: -P 1	Mo *K*α radiation, λ = 0.71073 Å
*a* = 10.635 (3) Å	Cell parameters from 3183 reflections
*b* = 10.888 (3) Å	θ = 3.2–27.5°
*c* = 11.310 (3) Å	µ = 0.36 mm^−^^1^
α = 96.838 (1)°	*T* = 153 K
β = 109.213 (1)°	Block, colourless
γ = 116.017 (3)°	0.47 × 0.45 × 0.41 mm
*V* = 1056.5 (5) Å^3^	

### Data collection

**Table:**

Rigaku AFC10/Saturn724+ diffractometer	4752 independent reflections
Radiation source: Rotating Anode	3984 reflections with *I* > 2σ(*I*)
Graphite monochromator	*R*_int_ = 0.021
Detector resolution: 28.5714 pixels mm^-1^	θ_max_ = 27.5°, θ_min_ = 3.2°
phi and ω scans	*h* = −13→13
Absorption correction: multi-scan (*SADABS*; Sheldrick, 1996)	*k* = −14→9
*T*_min_ = 0.848, *T*_max_ = 0.866	*l* = −14→14
10358 measured reflections	

### Refinement

**Table:**

Refinement on *F*^2^	Primary atom site location: structure-invariant direct methods
Least-squares matrix: full	Secondary atom site location: difference Fourier map
*R*[*F*^2^ > 2σ(*F*^2^)] = 0.034	Hydrogen site location: inferred from neighbouring sites
*wR*(*F*^2^) = 0.082	H-atom parameters constrained
*S* = 1.00	*w* = 1/[σ^2^(*F*_o_^2^) + (0.0339*P*)^2^ + 0.563*P*] where *P* = (*F*_o_^2^ + 2*F*_c_^2^)/3
4752 reflections	(Δ/σ)_max_ = 0.001
300 parameters	Δρ_max_ = 0.48 e Å^−^^3^
0 restraints	Δρ_min_ = −0.26 e Å^−^^3^

### Special details

**Table:**

Geometry. All e.s.d.'s (except the e.s.d. in the dihedral angle between two l.s. planes) are estimated using the full covariance matrix. The cell e.s.d.'s are taken into account individually in the estimation of e.s.d.'s in distances, angles and torsion angles; correlations between e.s.d.'s in cell parameters are only used when they are defined by crystal symmetry. An approximate (isotropic) treatment of cell e.s.d.'s is used for estimating e.s.d.'s involving l.s. planes.
Refinement. Refinement of *F*^2^ against ALL reflections. The weighted *R*-factor *wR* and goodness of fit *S* are based on *F*^2^, conventional *R*-factors *R* are based on *F*, with *F* set to zero for negative *F*^2^. The threshold expression of *F*^2^ > σ(*F*^2^) is used only for calculating *R*-factors(gt) *etc*. and is not relevant to the choice of reflections for refinement. *R*-factors based on *F*^2^ are statistically about twice as large as those based on *F*, and *R*- factors based on ALL data will be even larger.

### Fractional atomic coordinates and isotropic or equivalent isotropic displacement parameters (Å^2^)

**Table:**

	*x*	*y*	*z*	*U*_iso_*/*U*_eq_	
Cl1	1.02347 (5)	0.66238 (5)	0.52667 (4)	0.03025 (12)	
Cl2	1.55188 (5)	1.10870 (5)	0.83855 (4)	0.03247 (12)	
F1	0.21518 (11)	0.21751 (10)	0.77572 (10)	0.0272 (2)	
F2	−0.13376 (10)	0.31772 (11)	0.86694 (10)	0.0277 (2)	
F3	1.40442 (12)	1.12878 (12)	1.01131 (10)	0.0387 (3)	
O1	0.86955 (11)	0.70469 (12)	0.83236 (10)	0.0182 (2)	
O2	0.79299 (12)	0.66272 (12)	0.61291 (10)	0.0207 (2)	
O3	0.80388 (12)	0.93820 (11)	0.80944 (11)	0.0211 (2)	
N1	1.27582 (15)	0.88489 (14)	0.69998 (13)	0.0203 (3)	
N2	0.61806 (14)	0.82558 (13)	0.59926 (12)	0.0164 (3)	
C1	0.17396 (17)	0.31502 (16)	0.80292 (15)	0.0170 (3)	
C2	0.03987 (17)	0.26486 (17)	0.82120 (15)	0.0195 (3)	
H2	−0.0188	0.1676	0.8171	0.023*	
C3	−0.00431 (17)	0.36277 (18)	0.84561 (15)	0.0195 (3)	
C4	0.07763 (17)	0.50429 (17)	0.85192 (15)	0.0197 (3)	
H4	0.0426	0.5687	0.8672	0.024*	
C5	0.21328 (17)	0.55015 (17)	0.83529 (14)	0.0176 (3)	
H5	0.2719	0.6480	0.8408	0.021*	
C6	0.26640 (16)	0.45728 (16)	0.81069 (14)	0.0148 (3)	
C7	0.41797 (16)	0.51242 (15)	0.80320 (13)	0.0144 (3)	
C8	0.51496 (17)	0.45898 (16)	0.85426 (14)	0.0161 (3)	
H8	0.4797	0.3794	0.8864	0.019*	
C9	0.66232 (17)	0.52086 (16)	0.85858 (14)	0.0165 (3)	
H9	0.7268	0.4825	0.8915	0.020*	
C10	0.71436 (16)	0.63841 (16)	0.81465 (14)	0.0154 (3)	
C11	0.62084 (16)	0.69371 (15)	0.76086 (14)	0.0146 (3)	
C12	0.47150 (16)	0.62775 (16)	0.75432 (14)	0.0150 (3)	
H12	0.4048	0.6622	0.7157	0.018*	
C13	0.89366 (17)	0.71843 (16)	0.72222 (15)	0.0160 (3)	
C14	1.06198 (17)	0.81361 (16)	0.75919 (15)	0.0157 (3)	
C15	1.12842 (17)	0.79897 (16)	0.67455 (15)	0.0175 (3)	
C16	1.36315 (17)	0.99312 (17)	0.81058 (16)	0.0207 (3)	
C17	1.30851 (18)	1.01633 (17)	0.90099 (16)	0.0231 (3)	
C18	1.15754 (18)	0.92607 (17)	0.87740 (16)	0.0204 (3)	
H18	1.1194	0.9399	0.9398	0.024*	
C19	0.68695 (16)	0.82890 (15)	0.72380 (14)	0.0148 (3)	
C20	0.49642 (17)	0.69441 (16)	0.48941 (14)	0.0173 (3)	
H20A	0.4784	0.6097	0.5207	0.021*	
H20B	0.5316	0.6862	0.4195	0.021*	
C21	0.34556 (19)	0.69431 (19)	0.43204 (17)	0.0252 (4)	
H21A	0.3094	0.7008	0.5006	0.038*	
H21B	0.2675	0.6052	0.3594	0.038*	
H21C	0.3624	0.7769	0.3992	0.038*	
C22	0.69372 (18)	0.95545 (17)	0.56353 (16)	0.0220 (3)	
H22A	0.7312	1.0415	0.6357	0.026*	
H22B	0.6174	0.9539	0.4835	0.026*	
C23	0.8299 (2)	0.96539 (19)	0.53800 (18)	0.0288 (4)	
H23A	0.9051	0.9660	0.6169	0.043*	
H23B	0.8794	1.0541	0.5168	0.043*	
H23C	0.7923	0.8824	0.4640	0.043*	

### Atomic displacement parameters (Å^2^)

**Table:**

	*U*^11^	*U*^22^	*U*^33^	*U*^12^	*U*^13^	*U*^23^
Cl1	0.0241 (2)	0.0259 (2)	0.0298 (2)	0.00427 (17)	0.01557 (17)	−0.00399 (17)
Cl2	0.01363 (19)	0.0355 (2)	0.0353 (2)	0.00161 (17)	0.01171 (17)	0.00979 (19)
F1	0.0238 (5)	0.0163 (5)	0.0400 (6)	0.0075 (4)	0.0168 (5)	0.0064 (4)
F2	0.0139 (5)	0.0356 (6)	0.0319 (5)	0.0079 (4)	0.0141 (4)	0.0120 (4)
F3	0.0237 (5)	0.0331 (6)	0.0292 (6)	−0.0039 (5)	0.0099 (5)	−0.0086 (5)
O1	0.0106 (5)	0.0242 (6)	0.0184 (5)	0.0064 (4)	0.0071 (4)	0.0093 (4)
O2	0.0155 (5)	0.0233 (6)	0.0194 (5)	0.0079 (5)	0.0071 (4)	0.0040 (4)
O3	0.0162 (5)	0.0171 (5)	0.0195 (5)	0.0016 (4)	0.0057 (4)	0.0052 (4)
N1	0.0166 (6)	0.0224 (7)	0.0238 (7)	0.0089 (6)	0.0113 (5)	0.0093 (5)
N2	0.0156 (6)	0.0136 (6)	0.0177 (6)	0.0057 (5)	0.0066 (5)	0.0064 (5)
C1	0.0158 (7)	0.0162 (7)	0.0175 (7)	0.0073 (6)	0.0068 (6)	0.0049 (6)
C2	0.0141 (7)	0.0170 (7)	0.0178 (7)	0.0010 (6)	0.0059 (6)	0.0054 (6)
C3	0.0097 (7)	0.0278 (8)	0.0165 (7)	0.0055 (6)	0.0057 (6)	0.0080 (6)
C4	0.0152 (7)	0.0252 (8)	0.0211 (7)	0.0118 (7)	0.0076 (6)	0.0095 (6)
C5	0.0145 (7)	0.0184 (7)	0.0181 (7)	0.0066 (6)	0.0062 (6)	0.0087 (6)
C6	0.0115 (7)	0.0166 (7)	0.0120 (6)	0.0041 (6)	0.0040 (5)	0.0054 (5)
C7	0.0115 (7)	0.0148 (7)	0.0121 (6)	0.0035 (6)	0.0045 (5)	0.0034 (5)
C8	0.0162 (7)	0.0148 (7)	0.0144 (7)	0.0050 (6)	0.0067 (6)	0.0066 (5)
C9	0.0151 (7)	0.0192 (7)	0.0157 (7)	0.0092 (6)	0.0061 (6)	0.0066 (6)
C10	0.0101 (6)	0.0176 (7)	0.0144 (7)	0.0038 (6)	0.0057 (5)	0.0046 (6)
C11	0.0142 (7)	0.0137 (7)	0.0134 (6)	0.0047 (6)	0.0065 (5)	0.0042 (5)
C12	0.0136 (7)	0.0158 (7)	0.0142 (7)	0.0067 (6)	0.0053 (5)	0.0053 (5)
C13	0.0159 (7)	0.0146 (7)	0.0205 (7)	0.0085 (6)	0.0092 (6)	0.0075 (6)
C14	0.0137 (7)	0.0160 (7)	0.0200 (7)	0.0079 (6)	0.0085 (6)	0.0088 (6)
C15	0.0178 (7)	0.0166 (7)	0.0193 (7)	0.0087 (6)	0.0092 (6)	0.0065 (6)
C16	0.0134 (7)	0.0222 (8)	0.0257 (8)	0.0067 (6)	0.0095 (6)	0.0113 (6)
C17	0.0179 (8)	0.0198 (8)	0.0208 (8)	0.0034 (6)	0.0065 (6)	0.0018 (6)
C18	0.0187 (8)	0.0227 (8)	0.0207 (7)	0.0091 (7)	0.0115 (6)	0.0072 (6)
C19	0.0124 (7)	0.0150 (7)	0.0184 (7)	0.0065 (6)	0.0086 (6)	0.0061 (6)
C20	0.0174 (7)	0.0163 (7)	0.0154 (7)	0.0071 (6)	0.0061 (6)	0.0045 (6)
C21	0.0187 (8)	0.0245 (8)	0.0250 (8)	0.0097 (7)	0.0037 (7)	0.0054 (7)
C22	0.0215 (8)	0.0173 (8)	0.0229 (8)	0.0067 (7)	0.0077 (6)	0.0109 (6)
C23	0.0278 (9)	0.0266 (9)	0.0302 (9)	0.0072 (7)	0.0178 (8)	0.0141 (7)

### Geometric parameters (Å, º)

**Table:**

Cl1—C15	1.7255 (16)	C8—C9	1.389 (2)
Cl2—C16	1.7274 (16)	C8—H8	0.9500
F1—C1	1.3540 (18)	C9—C10	1.379 (2)
F2—C3	1.3596 (17)	C9—H9	0.9500
F3—C17	1.3413 (18)	C10—C11	1.391 (2)
O1—C13	1.3641 (18)	C11—C12	1.398 (2)
O1—C10	1.4101 (17)	C11—C19	1.507 (2)
O2—C13	1.1929 (18)	C12—H12	0.9500
O3—C19	1.2391 (18)	C13—C14	1.493 (2)
N1—C16	1.319 (2)	C14—C18	1.394 (2)
N1—C15	1.330 (2)	C14—C15	1.396 (2)
N2—C19	1.3444 (19)	C16—C17	1.380 (2)
N2—C20	1.4682 (19)	C17—C18	1.373 (2)
N2—C22	1.4704 (19)	C18—H18	0.9500
C1—C2	1.383 (2)	C20—C21	1.519 (2)
C1—C6	1.396 (2)	C20—H20A	0.9900
C2—C3	1.374 (2)	C20—H20B	0.9900
C2—H2	0.9500	C21—H21A	0.9800
C3—C4	1.376 (2)	C21—H21B	0.9800
C4—C5	1.391 (2)	C21—H21C	0.9800
C4—H4	0.9500	C22—C23	1.529 (2)
C5—C6	1.399 (2)	C22—H22A	0.9900
C5—H5	0.9500	C22—H22B	0.9900
C6—C7	1.490 (2)	C23—H23A	0.9800
C7—C8	1.397 (2)	C23—H23B	0.9800
C7—C12	1.397 (2)	C23—H23C	0.9800
			
C13—O1—C10	116.40 (11)	O1—C13—C14	110.21 (12)
C16—N1—C15	117.65 (13)	C18—C14—C15	117.25 (14)
C19—N2—C20	124.77 (13)	C18—C14—C13	120.94 (13)
C19—N2—C22	117.30 (12)	C15—C14—C13	121.72 (14)
C20—N2—C22	116.24 (12)	N1—C15—C14	124.14 (14)
F1—C1—C2	116.52 (13)	N1—C15—Cl1	114.07 (11)
F1—C1—C6	119.33 (13)	C14—C15—Cl1	121.77 (12)
C2—C1—C6	124.15 (15)	N1—C16—C17	122.56 (14)
C3—C2—C1	116.65 (14)	N1—C16—Cl2	117.10 (12)
C3—C2—H2	121.7	C17—C16—Cl2	120.33 (13)
C1—C2—H2	121.7	F3—C17—C18	120.62 (15)
F2—C3—C2	118.14 (14)	F3—C17—C16	119.09 (14)
F2—C3—C4	118.66 (15)	C18—C17—C16	120.29 (15)
C2—C3—C4	123.19 (14)	C17—C18—C14	118.05 (14)
C3—C4—C5	117.96 (15)	C17—C18—H18	121.0
C3—C4—H4	121.0	C14—C18—H18	121.0
C5—C4—H4	121.0	O3—C19—N2	122.80 (14)
C4—C5—C6	122.29 (14)	O3—C19—C11	118.38 (13)
C4—C5—H5	118.9	N2—C19—C11	118.82 (13)
C6—C5—H5	118.9	N2—C20—C21	111.98 (13)
C1—C6—C5	115.73 (14)	N2—C20—H20A	109.2
C1—C6—C7	123.36 (14)	C21—C20—H20A	109.2
C5—C6—C7	120.78 (13)	N2—C20—H20B	109.2
C8—C7—C12	118.48 (13)	C21—C20—H20B	109.2
C8—C7—C6	121.26 (13)	H20A—C20—H20B	107.9
C12—C7—C6	120.05 (13)	C20—C21—H21A	109.5
C9—C8—C7	120.72 (14)	C20—C21—H21B	109.5
C9—C8—H8	119.6	H21A—C21—H21B	109.5
C7—C8—H8	119.6	C20—C21—H21C	109.5
C10—C9—C8	119.46 (14)	H21A—C21—H21C	109.5
C10—C9—H9	120.3	H21B—C21—H21C	109.5
C8—C9—H9	120.3	N2—C22—C23	111.44 (14)
C9—C10—C11	121.79 (13)	N2—C22—H22A	109.3
C9—C10—O1	116.95 (13)	C23—C22—H22A	109.3
C11—C10—O1	121.19 (13)	N2—C22—H22B	109.3
C10—C11—C12	117.89 (13)	C23—C22—H22B	109.3
C10—C11—C19	119.70 (13)	H22A—C22—H22B	108.0
C12—C11—C19	122.16 (13)	C22—C23—H23A	109.5
C7—C12—C11	121.57 (14)	C22—C23—H23B	109.5
C7—C12—H12	119.2	H23A—C23—H23B	109.5
C11—C12—H12	119.2	C22—C23—H23C	109.5
O2—C13—O1	124.03 (14)	H23A—C23—H23C	109.5
O2—C13—C14	125.75 (14)	H23B—C23—H23C	109.5
			
F1—C1—C2—C3	−178.45 (13)	C10—O1—C13—C14	169.47 (12)
C6—C1—C2—C3	1.5 (2)	O2—C13—C14—C18	147.58 (16)
C1—C2—C3—F2	−178.57 (13)	O1—C13—C14—C18	−31.7 (2)
C1—C2—C3—C4	0.2 (2)	O2—C13—C14—C15	−28.9 (2)
F2—C3—C4—C5	177.39 (13)	O1—C13—C14—C15	151.77 (14)
C2—C3—C4—C5	−1.3 (2)	C16—N1—C15—C14	−2.1 (2)
C3—C4—C5—C6	1.0 (2)	C16—N1—C15—Cl1	179.14 (12)
F1—C1—C6—C5	178.17 (13)	C18—C14—C15—N1	0.1 (2)
C2—C1—C6—C5	−1.7 (2)	C13—C14—C15—N1	176.77 (14)
F1—C1—C6—C7	−6.0 (2)	C18—C14—C15—Cl1	178.85 (12)
C2—C1—C6—C7	174.07 (14)	C13—C14—C15—Cl1	−4.5 (2)
C4—C5—C6—C1	0.4 (2)	C15—N1—C16—C17	2.3 (2)
C4—C5—C6—C7	−175.48 (13)	C15—N1—C16—Cl2	−177.03 (12)
C1—C6—C7—C8	−33.6 (2)	N1—C16—C17—F3	179.96 (15)
C5—C6—C7—C8	142.01 (15)	Cl2—C16—C17—F3	−0.7 (2)
C1—C6—C7—C12	151.76 (14)	N1—C16—C17—C18	−0.6 (3)
C5—C6—C7—C12	−32.6 (2)	Cl2—C16—C17—C18	178.67 (13)
C12—C7—C8—C9	1.2 (2)	F3—C17—C18—C14	178.05 (15)
C6—C7—C8—C9	−173.54 (13)	C16—C17—C18—C14	−1.3 (3)
C7—C8—C9—C10	1.5 (2)	C15—C14—C18—C17	1.6 (2)
C8—C9—C10—C11	−2.7 (2)	C13—C14—C18—C17	−175.12 (15)
C8—C9—C10—O1	174.41 (12)	C20—N2—C19—O3	169.20 (14)
C13—O1—C10—C9	124.95 (14)	C22—N2—C19—O3	4.6 (2)
C13—O1—C10—C11	−57.97 (18)	C20—N2—C19—C11	−10.0 (2)
C9—C10—C11—C12	1.1 (2)	C22—N2—C19—C11	−174.58 (13)
O1—C10—C11—C12	−175.89 (13)	C10—C11—C19—O3	−55.73 (19)
C9—C10—C11—C19	175.39 (14)	C12—C11—C19—O3	118.36 (16)
O1—C10—C11—C19	−1.6 (2)	C10—C11—C19—N2	123.49 (15)
C8—C7—C12—C11	−2.8 (2)	C12—C11—C19—N2	−62.43 (19)
C6—C7—C12—C11	171.97 (13)	C19—N2—C20—C21	117.76 (16)
C10—C11—C12—C7	1.7 (2)	C22—N2—C20—C21	−77.50 (17)
C19—C11—C12—C7	−172.46 (13)	C19—N2—C22—C23	78.92 (17)
C10—O1—C13—O2	−9.8 (2)	C20—N2—C22—C23	−87.00 (16)

### Hydrogen-bond geometry (Å, º)

**Table:**

*D*—H···*A*	*D*—H	H···*A*	*D*···*A*	*D*—H···*A*
C2—H2···O3^i^	0.95	2.35	3.287 (2)	170

Symmetry code: (i) *x*−1, *y*−1, *z*.
